# Sulfolipids and glycolipid sulfotransferase activities in human renal cell carcinoma cells.

**DOI:** 10.1038/bjc.1993.12

**Published:** 1993-01

**Authors:** T. Kobayashi, K. Honke, K. Kamio, N. Sakakibara, S. Gasa, N. Miyao, T. Tsukamoto, I. Ishizuka, T. Miyazaki, A. Makita

**Affiliations:** Biochemistry Laboratory, Hokkaido University School of Medicine, Sapporo, Japan.

## Abstract

**Images:**


Br. J. Cancer (1993), 67, 76-80 © Macmillan Press Ltd., 1993

### Sulfolipids and glycolipid sulfotransferase activities in human renal cell carcinoma cells

T. Kobayashi[1], K. Honke[1], K. Kamio[1], N. Sakakibara[1], S. Gasa[1], N. Miyao[2], T. Tsukamoto[2], I. Ishizuka[3], T. Miyazaki[4] & A. Makita[1]

[1]*Biochemistry Laboratory, Cancer Institute, and* [4]*The 3rd Depertment of Internal Medicine, Hokkaido University School of Medicine, Sapporo, and* [2]*Department of Urology, Sapporo Medical College, Sapporo, and* [3]*Depertment of Biochemistry, Teikyo University, Tokyo, Japan.*

**Summary** A cell line (SMKT-R3) established from human renal cell carcinoma was characterised for the presence of sulfolipids and glycolipid sulfotransferases. Sulfolipids were found to constitute a large part of the acidic glycolipid fraction in SMKT-R3 cells. These findings were confirmed by metabolic labelling with $^{35}$S-sulfate. These sulfolipids were expressed at the surface of SMKT-R3 cells as ascertained by cytofluorometry using a monoclonal antibody directed to sulfolipids. Furthermore, markedly high activity levels of glycolipid sulfotransferases were observed in SMKT-R3 cells compared with other cell lines. These results suggest that the increased synthesis of sulfolipids in renal cell carcinoma tissue (Sakakibara *et al.*, 1989. *Cancer Res.*, 49, 335-339) is due to the elevation of the sulfotransferase activities of renal carcinoma cells themselves.

Glycolipids have been known to undergo marked cancer-associated changes (Hakomori, 1985). In particular, acidic glycolipids with sialic acid residues, called gangliosides, have been well studied. On the other hand, reports on cancer-associated changes of the other acidic glycolipids, sulfolipids, which contain sulfate residues, are relatively rare (Siddiqui *et al.*, 1978; Gasa *et al.*, 1979; Yoda *et al.*, 1979; Hattori *et al.*, 1981; Mitsuyama *et al.*, 1983; Hiraiwa *et al.*, 1988; Hiraiwa *et al.*, 1990). The synthesis of sulfolipids is catalysed by PAPS:GalCer sulfotransferase (EC 2.8.2.11) (Balasubramanian & Bachhawat, 1965). Although the sulfotransferase from rat kidney (Tennekoon *et al.*, 1985) and testis (Sakac *et al.*, 1992) has been recently purified, the human enzyme has not.

In our previous studies, sulfolipids were found to increase markedly in human renal cell carcinoma (Sakakibara *et al.*, 1989), but not in Wilms' tumour (Sakakibara *et al.*, 1991). The increment of the sulfolipid contents in renal cell carcinoma was associated with enhanced activity of glycolipid sulfotransferase in the cancer tissues (Sakakibara *et al.*, 1989). Furthermore, the level of the sulfotransferase appeared to be elevated in sera from patients with renal cell carcimona (Gasa *et al.*, 1990), and hepatocellular carcinoma (Gasa *et al.*, 1991).

Several cell lines established from mammalian kidney were characterised for sulfotransferase activity (Tadano & Ishizuka, 1979), but there has been no report on the enzyme activity and sulfolipids of renal cell carcinoma cells. This paper describes some properties of sulfolipids and glycolipid sulfotransferase activities in renal cell carcinoma cells.

### Materials and methods

### Materials

$^{35}$S-PAPS (1.5 Ci mmol$^{-1}$) and $^{35}$S-sodium sulfate (250-1000 mCi mmol$^{-1}$) were purchased from New England Nuclear; unlabeled PAPS and *p*-nitrocatechol sulfate from Sigma. DEAE-Sephadex A-25 and Sephadex G-25 were obtained from Pharmacia-LKB. GalCer and LacCer were purified in this laboratory from bovine brain and horse red cell membranes, respectively. Other reagents were of analytical grade.

Several human cell lines, A-431 (epidermoid carcinoma), PC-3 (lung adenocarcinoma), HL-60 (acute promyelocyte leukaemia), K-562 (chronic myelogenous leukaemia), were a gift from the Japanese Cancer Research Resources Bank.

### Cell culture

SMKT-R3 cells were established from human renal cell carcinoma as described previously (Miyao *et al.*, 1989), and cultured in Dulbecco's modified minimal essential medium supplemented with 10% foetal bovine serum.

### Preparation of glycolipids

Cell monolayers were washed with Tris-buffered saline and harvested by scraping with a rubber policeman. Then the cell suspensions were centrifuged and washed three times with Tris-buffered saline. The cell pellets (approximately 10 mg protein) were extracted with 50 ml of a mixture of chloroform/methanol/water (60:35:8, the ratio of the solvent mixture is expressed by volume), and then re-extracted with 50 ml of a mixture of chloroform/methanol/water (30:60:8). The two extracts were combined and subjected to mild alkaline hydrolysis to destroy ester lipids, followed by neutralisation with acetic acid. After evaporation of the solvent, the total lipid extract was desalted with a Sephadex G-25 column. The eluate was concentrated and applied to a DEAE-Sephadex A-25 (acetate form) column. After washing with chlorofom/methanol/water (30:60:8), the acidic glycolipid fraction was eluted with chloroform/methanol/1 M $CH_3COONH_4$ (30:60:8), evaporated, and desalted as above.

### Analysis of glycolipids

Glycolipids were chromatographed on precoated Silica Gel 60 HPTLC plates (Merck) using the solvent system: chloroform/methanol/0.2% $CaCl_2$ (60:35:7). Orcinol, resorcinol, and Azure A reagents were used for detection of hexose-containing glycolipids (Svennerholm, 1956), gangliosides (Svennerholm, 1957), and sulfolipids (Iida *et al.*, 1989), respectively. TLC-immunostaining was performed using an anti-sulfolipid monoclonal antibody, Sulph I (Fredman *et al.*, 1988), and peroxidase-conjugated sheep anti-mouse immunoglobulins as described previously (Magnani *et al.*, 1982).

Correspondence: K. Honke, Biochemistry Laboratory, Cancer Institute, Hokkaido University School of Medicine, Kita-ku N15 W7, Sapporo, Japan.

Abbreviations: PAPS, 3'-phosphoadenosine-5'-phosphosulfate; GalCer, galactosylceramide; GlcCer, glucosylceramide; LacCer, lactosylceramide; SB2, bis-sulfogangliotriaosylceramide; SM2, gangliotriaosylceramide 3'-sulfate; SM3, lactosylceramide 3'-sulfate; SM4, galactosylceramide 3'-sulfate; TLC, thin-layer chromatography.

Received 18 June 1992; and in revised form 10 August 1992.

*Metabolic labelling of SMKT-R3 cells*

Monolayer cultures of SMKT-R3 cells ($3 \times 10^6$ cells) were labelled with 5 $\mu$Ci $ml^{-1}$ $^{35}S$-sodium sulfate for 24 h. Labelled acidic glycolipids were prepared, chromatographed as described above, and detected by autoradiography.

*Assay of glycolipid sulfotransferase activities and identification of the reaction products*

The cell pellets, which were prepared as above, were resuspended in 10 mM Tris-HCl, pH 7.5, 150 mM NaCl, 0.1% Lubrol PX and sonicated on ice. Glycolipid sulfotransferase activities of the cell homogenate, the protein concentration of which was adjusted to approximately 1 mg $ml^{-1}$, were assayed using GalCer and LacCer separately as substrates by a previously described method (Kawano *et al.*, 1989). The minimal detectable level of the assay was 30–50 pmol $h^{-1}$ $mg^{-1}$ protein. The synthesised products were isolated, desalted, chromatographed on a TLC plate, and scanned for radioactivity, according to a previous procedure (Kawano *et al.*, 1989).

*Assay of arylsulfatase A activity*

Arylsulfatase A activity of the cell homogenate was assayed by the method of Baum *et al.* (1965).

*Cytofluorometric analysis*

SMKT-R3 cells were incubated for 24 h in the culture medium with or without 0.5 mM sodium selenate. The cells were harvested, washed, and stained by the indirect immunofluorescence method; the cells were reacted with Sulph I as the first antibody and subsequently with fluorescein isothiocyanate-conjugated $F(ab')_2$ fragment of rabbit anti-mouse immunoglobulins (DAKO) as the second antibody. Fluorescence profiles were determined with a FACScan (Becton Dickinson).

**Figure 2** Detection of cell surface expression of sulfolipids by flow cytometry. SMKT-R3 cells were cultured in the presence (lower) or absence (upper) of sodium selenate. The cells were reacted with the monoclonal antibody Sulph I, and fluorescein-conjugated $F(ab')_2$ to mouse IgG, followed by flow cytometry. The solid line indicates reactivity with Sulph I; the dotted line, reactivity with nonspecific isotype mouse IgG.

**Figure 1** Thin-layer chromatogram of acidic glycolipids from SMKT-R3 cells. O, origin. **a**, Lanes 1,3 and 5, sulfolipid standards: SM4, SM3, SM2 and SB2 from the top to the bottom; lanes 2, 4 and 5, acidic glycolipids from SMKT-R3 cells, each corresponding to 1 mg of cell protein. Glycolipids were chromatographed and visualised with an orcinol reagant (lanes 1 and 2), or with an azure A reagent (lanes 3 and 4) or by immunostaining (lanes 5 and 6) as described under 'Materials and methods'. **b**, Lane 1, sulfolipid standards stained with an orcinol reagent as shown in **a**. Lane 2, autoradiogram of a TLC plate of labelled SMKT-R3 cell lipids. The cells were metabolically labelled with $^{35}S$-sulfate. Acidic glycolipids extracted from the cells, corresponding to 300 $\mu$g of cell protein were chromatographed and detected by autoradiography. Minor sulfolipids are marked with asterisks.

### Results

*Acidic glycolipids from human renal cell carcinoma cells*

When acidic glycolipid fractions extracted from SMKT-R3 cells were analysed by TLC, a number of glycolipids were detected as shown in Figure 1a. Three of them, co-migrating with authentic SM4, SM3, and SM2, were found to be negative with resorcinol reagent (data not shown) but positive with Azure A reagent as well as with orcinol reagent. The monoclonal antibody Sulph I, which recognises non-reducing terminal galactose-3-O-sulfate (Fredman *et al.*, 1988), reacted specifically with the cell glycolipids consistent with standard SM4 and SM3. Taken together, the three glycolipids were identified as sulfolipids. These sulfolipids appeared as doublets, probably due to heterogeneity of the lipic moiety. Thus sulfolipids constituted a large part of the acidi glycolipid fraction in SMKT-R3 cells. These observations were confirmed by metabolic labelling with $^{35}$S-sulfate as shown in Figure 1b. Five sulfolipids were detected by autoradiography of the thin-layer chromatogram of the acidic glycolipid extract from the cells. In addition to the sulfolipids corresponding to reference SM4, SM3 and SM2, two minor, more slowing migrating sulfolipids (asterisks) were also detected, but they remain to be characterised.

*Cytofluorometric analysis of SMKT-R3 cells*

In order to ascertain sulfolipid expression on the cell surface, SMKT-R3 cells were analysed with a fluorescence-activated cell sorter using the monoclonal antibody Sulph I. As shown in Figure 2 (upper), Sulph I gave good cell surface reactivity with SMKT-R3 cells. Incubation of the cells with sodium selenate, which inhibits the synthesis of sulfolipids (Aruffo *et al.*, 1991), resulted in a reduction of expression of these sulfolipids (Figure 2 lower).

*Characterisation of glycolipid sulfotransferase of SMKT-R3 cells*

We previously established a rapid procedure for the determination of glycolipid sulfotransferase activity using rat kidney tissue as an enzyme source (Kawano *et al.*, 1989). SMKT-R3 cell homogenates were examined to detect the sulfotransferase activity through this assay procedure. When GalCer and LacCer were separately used as substrates, the products co-migrated with authentic SM4 and SM3, respectively, confirming the presence of glycolipid sulfotransferase activities and the validity of utilising this assay method (Figure 3). The effect of substrate concentration on the sulfotransferase activities of SMKT-R3 cells is shown in Figure 4. The *Km* values of the enzyme for GalCer and LacCer calculated from a Lineweaver-Burk plot were 43.2μM and 358μM, respectively.

**Figure 3** Characterisation of radiolabelled products of the glycolipid sulfotransferase reactions. Labelled sulfolipids synthesised by the sulfotransferases of SMKT-R3 cell homogenates were extracted, chromatographed on a thin-layer plate, and scanned for radioactivity. **a**, Sulfolipid standards stained with an orcinol reagent. **b**, Labelled product from GalCer substrate. **c**, Labelled product from LacCer substrate. O, origin.

**Figure 4** Effect of substrate concentrations on glycolipid sulfotransferase activities of SMKT-R3 cells. GalCer (●) and LacCer (○) were separately used as substrates. The obtained Lineweaver-Burk plots are shown.

*Glycolipid sulfotransferase and arylsulfatase A activities in various human cell lines*

Various human cell lines were evaluated to determine whether the sulfotransferase activities were characteristic of renal cell carcinoma cells or not. Interestingly, the sulfotransferase activities could not be detected in the cell lines other than SMKT-R3 under our assay conditions (Table I). The specific activities of glycolipid sulfotransferase toward GalCer and LacCer were 8690 pmol $h^{-1}$ $mg^{-1}$ protein and 3015 pmol $h^{-1}$ $mg^{-1}$ protein, respectively. On the other hand, the activities of arylsulfatase A, which catalyses hydrolysis of sulfolipids, were not significantly different in these cell lines (Table I). Therefore, it was suggested that the accumulation of sulfolipids in SMKT-R3 cells was due to their increased synthesis, and that the elevated sulfotransferase activities were unique to renal cell carcinoma cells.

### Discussion

In our previous study, a significantly elevated level of glycolipid sulfotransferases associated with accumulation of sulfolipids was demonstrated in human renal cell carcinoma tissues (Sakakibara *et al.*, 1989). These findings were confirmed and carried forward by the present study, where sulfolipids and glycolipid sulfotransferases were found to be expressed in renal cell carcinoma cells themselves.

The glycolipid patterns and the sulfotransferase activities of other human renal cell carcinoma cell lines, SMKT-R1 and SMKT-R2, (Miyao *et al.*, 1989) were similar to those of SMKT-R3 (data not shown). Glycolipid sulfotransferase activities could be detected only in renal cell carcinoma cell lines as far as we could examine, although there are reports documenting the expression of sulfolipids in other tumour cell lines including HL-60 cells (Hiraiwa *et al.*, 1988; Krivan *et al.*, 1989; Hiraiwa *et al.*, 1990; Aruffo *et al.*, 1991). When acidic glycolipid fractions from the other cell lines than the renal cell carcinoma cells were examined on TLC, sulfolipids could not be detected (data not shown). Our observations are consistent with the fact that the preferential expression of sulfated glycolipids is tissue-specific and relatively restricted to brain, kidney and small intestine (Makita & Taniguchi, 1985).

The specific activity of sulfotransferase towards GalCer in SMKT-R3 cells was 50-fold greater than that in normal

**Table 1** Activity levels of glycolipid sulfotransferases and arylsulfatase A in various human cell lines

| *Cell* | *Origin* | *Galactosyceramide sulfotransferase (pmol $h^{-1}$ $mg^{-1}$)* | *Lactosylceramide sulfotransferase (pmol $h^{-1}$ $mg^{-1}$)* | *Arylsulfatase A (nmol $h^{-1}$ $mg^{-1}$)* |
|---|---|---|---|---|
| SMKT-R3 | Renal cell carcinoma | 8690 | 3015 | 207 |
| PC-3 | Lung adenocarcinoma | N.D.[a] | N.D. | 128 |
| A-431 | Epidermoid carcinoma | N.D. | N.D. | 148 |
| HL-60 | Acute promyelocyte leukaemia | N.D. | N.D. | 110 |
| K-562 | Chronic myelogenous leukaemia | N.D. | N.D. | 224 |
| Fibroblast | Normal skin | N.D. | N.D. | 213 |

[a]N.D., Not detected.

human kidney tissue, and 8-fold greater than that in renal cell carcinoma tissue as shown in our previous report (Sakakibara *et al.*, 1989). Similar results were obtained as to LacCer sulfotransferase activity (Sakakibara *et al.*, 1989). Since human renal cell carcinoma is thought to originate from proximal tubular cells (Tannenbaum *et al.*, 1971), one of the reasons for the difference of the specific activities between renal cell carcinoma tissues and cells may be that the tissues contain other histological cells such as stromal cells that do not express the sulfotransferase activities. Furthermore, the specific activities for the sulfotransferase in SMKT-R3 cells were much greater than those in MDCK cells and JTC-12 cells, which were isolated from dog and monkey kidney, respectively (Tadano & Ishizuka, 1979), although the assay conditions were slightly different. Taken together, it is suggested that the sulfotransferase activities are characteristic of renal cells, and that the elevation of the enzymes is caused by the malignant changes of renal cells.

The sulfotransferase preparation from SMKT-R3 cells could sulfate GalCer and LacCer. Competition studies have suggested that GalCer and LacCer are sulfated by a single enzyme in MDCK cells and JTC-12 cells (Tadano & Ishizuka, 1979). Similar results were obtained from rat and boar testis sulfotransferases (Handa *et al.*, 1974; Lingwood, 1985). The *Km* value for GalCer of the sulfotransferase from SMKT-R3 cells was smaller than that for LacCer (Figure 4), in good concordance with those from MDCK cells and JTC-12 cells (Tadano & Ishizuka, 1979). Therefore, the sulfotransferase appears to prefer GalCer to LacCer as a substrate. In fact, the specific activity for GalCer was higher than that for LacCer in SMKT-R3 cells within a limited amount of substrate.

The SM3 content was much greater than that of SM4 (Figure 1), although more monohexosylceramides than dihexoslyceramides were contained in the neutral fraction of SMKT-R3 cells (data not shown). Human kidney contains GalCer and GlcCer as monohexosylceramide with a higher content of GlcCer, and the ratio of GlcCer in renal cell carcinoma tissues increases compared with that in uninvolved tissues (Saga *et al.*, 1990). Therefore, the amount of precursor glycolipids may regulate the synthesis of sulfolipids. One other explanation for the discrepancy may be that SM4 is more easily degraded by hydrolases, including arylsulfatase A, than SM3 in renal carcinoma cells.

Sulfolipids have been demonstrated to have a variety of biological interactions with extracellular matrix and blood coagulation modulators, etc. (Roberts, 1987). SMKT-R3 cells provide a useful model system for studying such sulfolipid functions as well as various aspects of glycolipid sulfotransferases and the sulfolipid metabolism in renal cancer cells.

We thank Ms M. Yamane for her research assistance and Mr K. Barrymore for his help in the preparation of this manuscript.

### References

ARUFFO, A., KOLANUS, W., WALZ, G., FREDMAN, P. & SEED, B. (1991). CD62/P-selectin recognition of myeloid and tumor cell sulfatides. *Cell*, **67**, 35–44.

BALASUBRAMANIAN, A.S. & BACHHAWAT, B.K. (1965). Studies on enzymic synthesis of cerebroside sulfate from 3'-phosphoadenosine 5'-phosphosulfate. *Indian J. Biochem.*, **2**, 212–216.

BAUM, H., DODGSON, K.S. & SPENCER, B. (1959). The assay of arylsulfatase A and B in human urine. *Clin. Chim. Acta*, **4**, 453–455.

FREDMAN, P., MATTSSON, L., ANDERSSON, K., DAVIDSSON, P., ISIZUKA, I., JEANSSON, S., MÅNSSON, J.-E. & SVENNERHOLM, L. (1988). Characterization of the binding epitope of a monoclonal antibody to sulphatide. *Biochem. J.*, **251**, 17–22.

GASA, S., MAKITA, A., HIRAMA, M. & KAWABATA, M. (1979). Cerebroside sulfotransferase activity in human lung tissues. *J. Biochem.*, **86**, 265–267.

GASA, S., CASL, M.-T., MAKITA, A., SAKAKIBARA, N., KOYANAGI, T. & ATSUTA, T. (1990). Presence and characterization of glycolipid sulfotransferase in human cancer serum. *Eur. J. Biochem.*, **189**, 301–306.

GASA, S., CASL, M-T., JIN, T., KAMIO, K., UEHARA, Y., MIYAZAKI, T. & MAKITA, A. (1991). Elevated serum level of glycolipid sulfotransferase in patients with hepatocellular carcinoma. *Cancer Lett.*, **59**, 19–24.

HAKOMORI, S. (1985). Aberrant glycosylation in cancer cell membranes as focused on glycolipids: overview and prospectives. *Cancer Res.*, **45**, 2405–2414.

HANDA, S., YAMATO, K., ISHIZUKA, I., SUZUKI, A. & YAMAKAWA, T. (1974). Biosynthesis of Seminolipid: sulfation *in vivo* and *in vitro*. *J. Biochem.*, **75**, 77–83.

HATTORI, H., UEMURA, K. & TAKETOMI, T. (1981). Glycolipids of gastric cancer. The presence of blood-group A-active glycolipids in cancer tissues from blood group patients. *Biochem. Biophys. Acta*, **666**, 361–369.

HIRAIWA, N., IIDA, N., ISHIZUKA, ITAI, S., SHIGETA, K., KANNAGI, R., FUKUDA, Y. & IMURA, H. (1988). Monoclonal antibodies directed to a disulfated glycosphingolipid, $S_{B1a}$($GgOse_4Cer$-$II^3IV^3$-bis-sulfate), associated with human hepatocellular carcinoma. *Cancer Res.*, **48**, 6769–6774.

HIRAIWA, N., FUKUDA, Y., IMURA, H., TADANO, A.K., NAGAI, K., ISHIZUKA, I. & KANNAGI, R. (1990). Accumulation of highly acidic sulfated glycosphingolipids in human hepatocellular carcincoma defined by a series of monoclonal antibodies. *Cancer Res.*, **50**, 2917–2928.

IIDA, N., TOIDA, T., KUSHI, Y., HANDA, S., FREDMAN, P., SVENNERHOLM, L. & ISHIZUKA, I. (1989). A sulfated glucosylceramide from rat kidney. *J. Biol. Chem.*, **264**, 5974–5980.

KAWANO, M., HONKE, K., TACHI, M., GASA, S. & MAKITA, A. (1989). An assay method for ganglioside synthase using anion-exchange chromatography. *Anal. Biochem.*, **182**, 9–15.

KRIVAN, H.C., OLSON, L.D., BARILE, M.F., GINSBURG, V. & ROBERTS, D.D. (1989). Adhesion of mycoplasma pneumoniae to sulfated glycolipids and inhibition by dextran sulfate. *J. Biol. Chem*, **264**, 9283–9288.

LINGWOOD, C.A. (1985). Developmental regulation of galactoglycerolipid and galactosphingolipid sulfation during mammalian spermatogenesis. *Biochem. J.*, **231**, 394–400.

MAGNANI, J.L., NILSSON, B., BROCKHAUS, M., ZOPF, D., STEPLEWSKI, Z., KOPROWSKI, H. & GINSVURG, V. (1982). A monoclonal antibody-defined antigen associated with gastrointestinal cancer is a ganglioside containing sialylated lacto-N-fucopentaose II. *J. Biol. Chem.*, **257**, 14365–14369.

MAKITA, A. & TANIFUCHI, N. (1985). Glycosphingolipids. In *New Comprehensive Biochemistry*, Wiegandt, H. (ed.) vol. 10, pp. 1–99. Elsevier: Amsterdam.

MITSUYAMA, T., GASA, S., TANIGUCHI, N., MAKITA, A., MIYASAKA, S., MATSUMURA, M., TSUKADA, M. & ISHIKURA, M. (1983). Elevation of sulfatide synthesis in human gastric adenocarcinoma: biochemical characteristics common to adenocarcinomas. *J. Exp. Clin. Cancer Res.*, **2**, 25–30.

MIYAO, N., TSUKAMOTO, T. & KUMAMOTO, Y. (1989). Establishment of three human renal cell carcinoma cell lines (SMKT-R-1, SMKT-R-2, and SMKT-R-3) and their characters. *Urol. Res.*, **17**, 317–324.

ROBERTS, D.D. (1987). Sulfatide-binding proteins. In *Methods in Enzymology*, Ginsburg, V. (ed.) vol. 138, pp. 473–483. Academic Press: New York.

SAGA, Y., GASA, S., MAKITA, A. & OIKAWA, K. (1990). Analytical and preparative separation of glucosylceramide and galactosylceramide by borate-impregnated silica gel chromatography. *J. Chromat.*, **513**, 379–383.

SAKAC, D., ZACHOS, M. & LINGWOOD, C.W. (1992). Purification of the testicular galactolipid: 3′-phosphoadenosine 5′-phosphosulfate sulfotransferase. *J. Biol. Chem.*, **267**, 1655–1659.

SAKAKIBARA, N., GASA, S., KAMIO, K., MAKITA, A. & KOYANAGI, T. (1989). Association of elevated sulfatides and sulfotransferase activities with human renal cell carcinoma. *Cancer Res.*, **49**, 335–339.

SAKAKIBARA, N., GASA, S., KAMIO, K., MAKITA, A., NONOMURA, K., TOGASHI, M., KOYANAGI, T., HATAE, Y. & TAKEDA, K. (1991). Distinctive glycolipid patterns in Wilms; tumor and renal cell carcinoma. *Cancer Lett.*, **57**, 187–192.

SIDDIQUI, B., WHITEHEAD, J.S. & KIM, Y.S. (1978). Glycoshingolipids in colonic adenocarcinoma. *J. Biol. Chem.*, **253**, 2168–2175.

SVENNERHOLM, L. (1956). The quantitative estimation of cerebrosides in nervous tissue. J. Neurochem., **1**, 42–53.

SVENNERHOLM, L. (1957). Quantitative estimation of sialic acid. II. A colorimetric resolcinol-hydrochloric acid method. *Biochim. Biophys. Acta*, **24**, 604–611.

TADANO, K. & ISHIZUKA, I. (1979). Enzymatic sulfation of galactosylceramides in cell lines derived from renal tubules. *Biochim. Biophys. Acta*, **575**, 421–430.

TANNENBAUM, M. (1971). Ultrastructural pathology of human renal cell tumors. *Pathol. Annu.*, **6**, 249–277.

TENNEKOON, G., AITCHISON, S. & ZARUBA, M. (1985). Purification and characterization of galactocerebroside sulfotransferase from rat kidney. *Arch. Biochem. Biophys.*, **240**, 932–944.

YODA, Y., GASA, S., MAKITA, A., FUJIOKA, Y., KIKUCHI, Y. & HASHIMOTO, M. (1979). Glycolipids in human lung carcinoma of histologically different types. *J. Natl. Cancer Inst.*, **63**, 1153–1160.


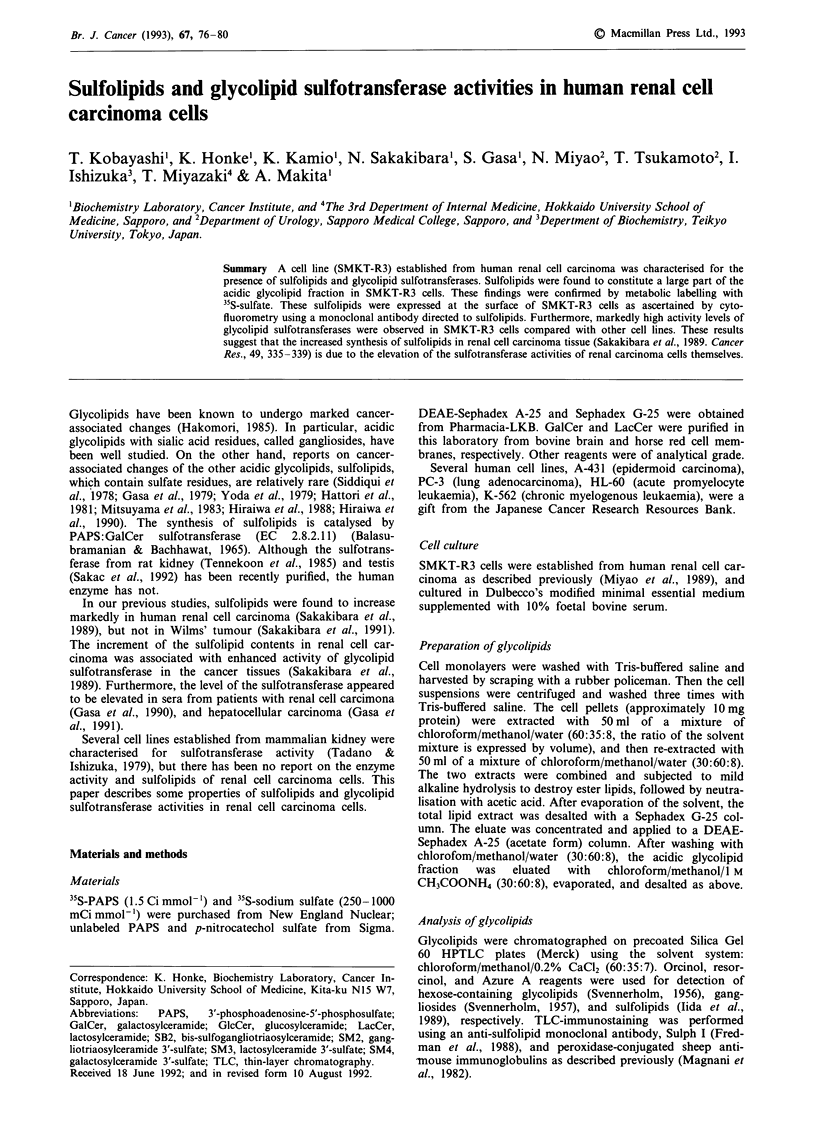

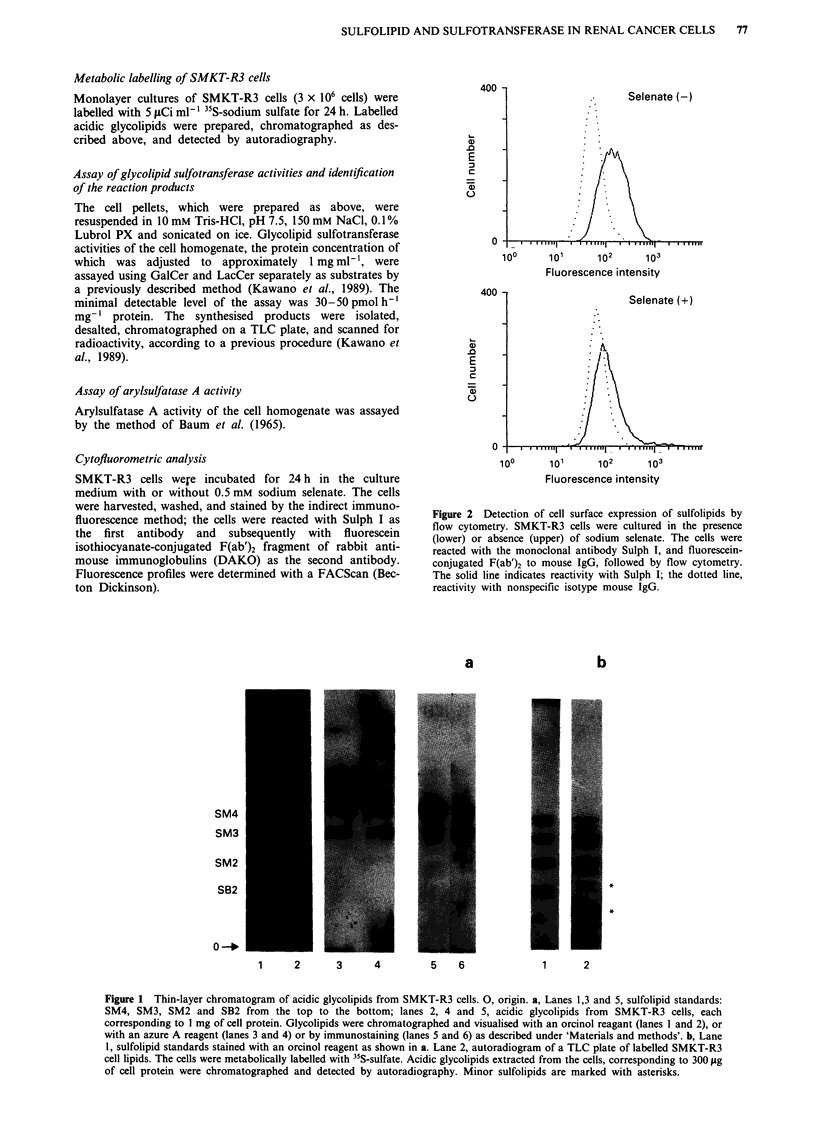

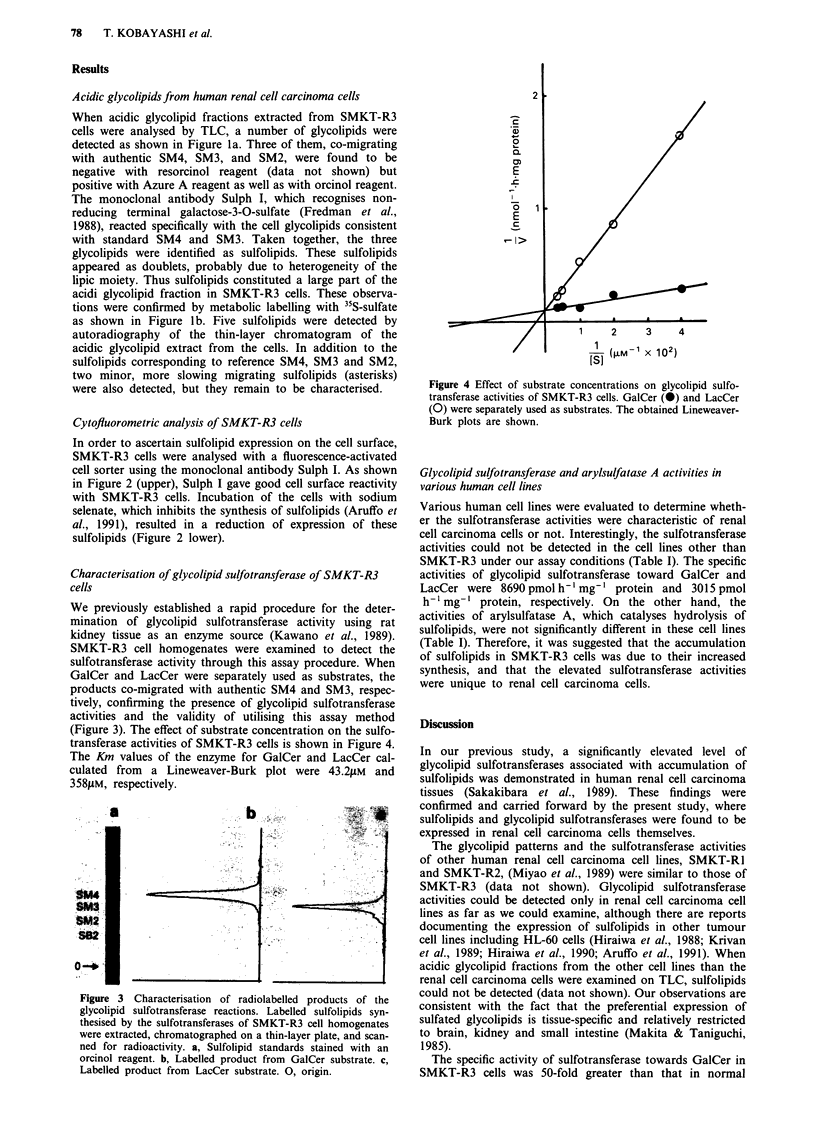

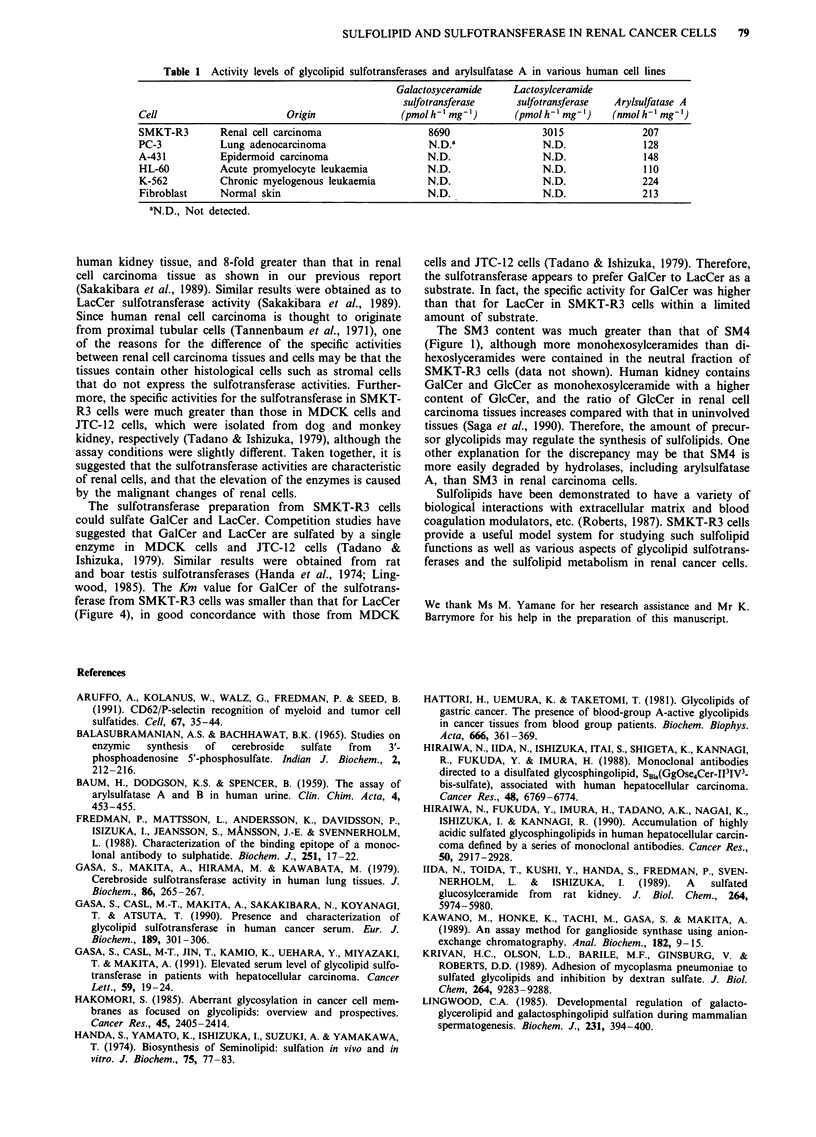

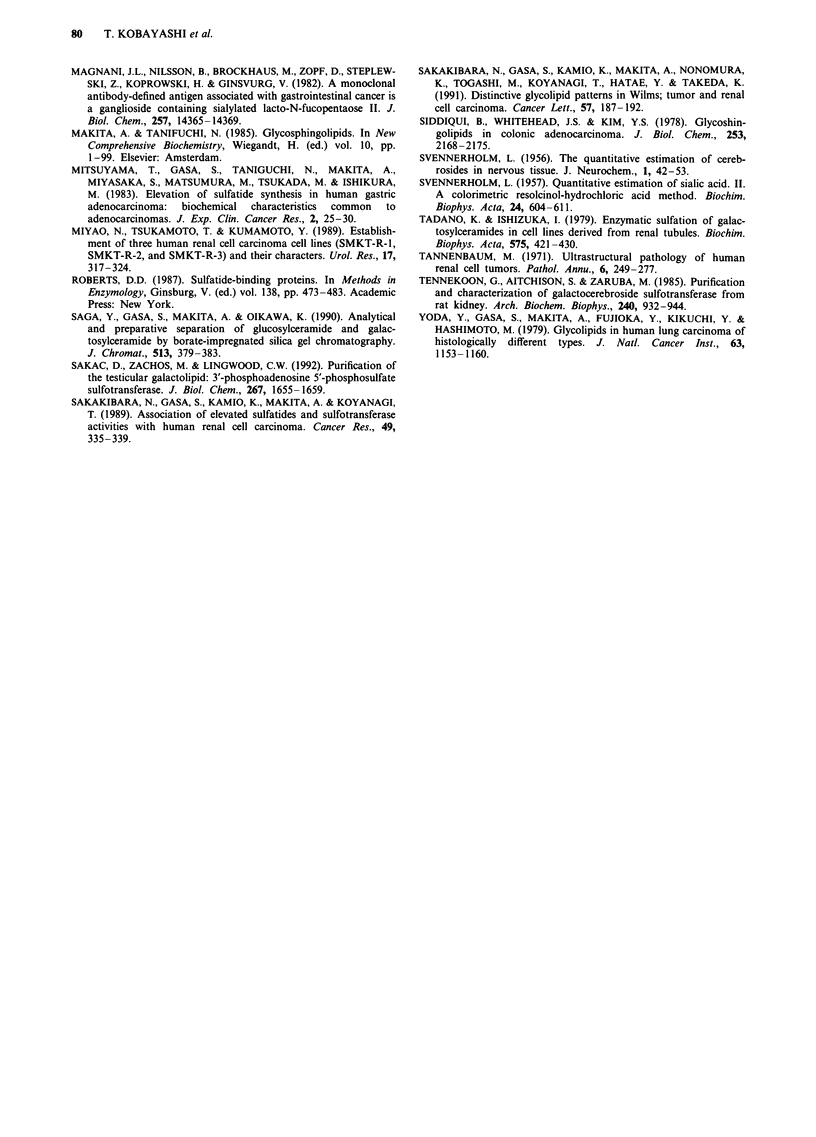


## References

[OCR_00488] Aruffo A., Kolanus W., Walz G., Fredman P., Seed B. (1991). CD62/P-selectin recognition of myeloid and tumor cell sulfatides.. Cell.

[OCR_00499] BAUM H., DODGSON K. S., SPENCER B. (1959). The assay of arylsulphatases A and B in human urine.. Clin Chim Acta.

[OCR_00493] Balasubramanian A. S., Bachhawat B. K. (1965). Studies on enzymic synthesis of cerebroside sulphate from 3'-phosphoadenosine-5'-phosphosulphate.. Indian J Biochem.

[OCR_00504] Fredman P., Mattsson L., Andersson K., Davidsson P., Ishizuka I., Jeansson S., Månsson J. E., Svennerholm L. (1988). Characterization of the binding epitope of a monoclonal antibody to sulphatide.. Biochem J.

[OCR_00521] Gasa S., Casl M. T., Jin T., Kamio K., Uehara Y., Miyazaki T., Makita A. (1991). Elevated serum level of glycolipid sulfotransferase in patients with hepatocellular carcinoma.. Cancer Lett.

[OCR_00515] Gasa S., Casl M. T., Makita A., Sakakibara N., Koyanagi T., Atsuta T. (1990). Presence and characterization of glycolipid sulfotransferase in human cancer serum.. Eur J Biochem.

[OCR_00510] Gasa S., Makita A., Hirama M., Kawabata M. (1979). Cerebroside sulfotransferase activity in human lung tissues. An elevated level in lung adenocarcinoma.. J Biochem.

[OCR_00527] Hakomori S. (1985). Aberrant glycosylation in cancer cell membranes as focused on glycolipids: overview and perspectives.. Cancer Res.

[OCR_00532] Handa S., Yamato K., Ishizuka I., Suzuki A., Yamakawa T. (1974). Biosynthesis of seminolipid: sulfation in vivo and in vitro.. J Biochem.

[OCR_00537] Hattori H., Uemura K., Taketomi T. (1981). The presence of blood group A-active glycolipids in cancer tissues from blood group O patients.. Biochim Biophys Acta.

[OCR_00550] Hiraiwa N., Fukuda Y., Imura H., Tadano-Aritomi K., Nagai K., Ishizuka I., Kannagi R. (1990). Accumulation of highly acidic sulfated glycosphingolipids in human hepatocellular carcinoma defined by a series of monoclonal antibodies.. Cancer Res.

[OCR_00543] Hiraiwa N., Iida N., Ishizuka I., Itai S., Shigeta K., Kannagi R., Fukuda Y., Imura H. (1988). Monoclonal antibodies directed to a disulfated glycosphingolipid, SB1a (GgOse4Cer-II3IV3-bis-sulfate), associated with human hepatocellular carcinoma.. Cancer Res.

[OCR_00559] Iida N., Toida T., Kushi Y., Handa S., Fredman P., Svennerholm L., Ishizuka I. (1989). A sulfated glucosylceramide from rat kidney.. J Biol Chem.

[OCR_00563] Kawano M., Honke K., Tachi M., Gasa S., Makita A. (1989). An assay method for ganglioside synthase using anion-exchange chromatography.. Anal Biochem.

[OCR_00568] Krivan H. C., Olson L. D., Barile M. F., Ginsburg V., Roberts D. D. (1989). Adhesion of Mycoplasma pneumoniae to sulfated glycolipids and inhibition by dextran sulfate.. J Biol Chem.

[OCR_00574] Lingwood C. A. (1985). Developmental regulation of galactoglycerolipid and galactosphingolipid sulphation during mammalian spermatogenesis. Evidence for a substrate-selective inhibitor of testicular sulphotransferase activity in the rat.. Biochem J.

[OCR_00583] Magnani J. L., Nilsson B., Brockhaus M., Zopf D., Steplewski Z., Koprowski H., Ginsburg V. (1982). A monoclonal antibody-defined antigen associated with gastrointestinal cancer is a ganglioside containing sialylated lacto-N-fucopentaose II.. J Biol Chem.

[OCR_00600] Miyao N., Tsukamoto T., Kumamoto Y. (1989). Establishment of three human renal cell carcinoma cell lines (SMKT-R-1, SMKT-R-2, and SMKT-R-3) and their characters.. Urol Res.

[OCR_00606] Roberts D. D. (1987). Sulfatide-binding proteins.. Methods Enzymol.

[OCR_00643] SVENNERHOLM L. (1957). Quantitative estimation of sialic acids. II. A colorimetric resorcinol-hydrochloric acid method.. Biochim Biophys Acta.

[OCR_00639] SVENNERHOLM L. (1956). The quantitative estimation of cerebrosides in nervous tissue.. J Neurochem.

[OCR_00617] Sakac D., Zachos M., Lingwood C. A. (1992). Purification of the testicular galactolipid: 3'-phosphoadenosine 5'-phosphosulfate sulfotransferase.. J Biol Chem.

[OCR_00622] Sakakibara N., Gasa S., Kamio K., Makita A., Koyanagi T. (1989). Association of elevated sulfatides and sulfotransferase activities with human renal cell carcinoma.. Cancer Res.

[OCR_00628] Sakakibara N., Gasa S., Kamio K., Makita A., Nonomura K., Togashi M., Koyanagi T., Hatae Y., Takeda K. (1991). Distinctive glycolipid patterns in Wilms' tumor and renal cell carcinoma.. Cancer Lett.

[OCR_00634] Siddiqui B., Whitehead J. S., Kim Y. S. (1978). Glycosphingolipids in human colonic adenocarcinoma.. J Biol Chem.

[OCR_00648] Tadano K., Ishizuka I. (1979). Enzymatic sulfation of galactosyl- and lactosylceramides in cell lines derived from renal tubules.. Biochim Biophys Acta.

[OCR_00653] Tannenbaum M. (1971). Ultrastructural pathology of human renal cell tumors.. Pathol Annu.

[OCR_00657] Tennekoon G., Aitchison S., Zaruba M. (1985). Purification and characterization of galactocerebroside sulfotransferase from rat kidney.. Arch Biochem Biophys.

[OCR_00662] Yoda Y., Gasa S., Makita A., Fujioka Y., Kikuchi Y., Hashimoto M. (1979). Glycolipids in human lung carcinoma of histologically different types.. J Natl Cancer Inst.

